# Conformational transitions of *Streptococcus pyogenes* Cas9 induced by salt and single-guide RNA binding

**DOI:** 10.1016/j.jbc.2024.108120

**Published:** 2024-12-21

**Authors:** Yufan He, Nikita Zalenski, Anthony A. Stephenson, Austin T. Raper, Chiran Ghimire, Zucai Suo

**Affiliations:** 1 Department of Biomedical Sciences, College of Medicine, Florida State University, Tallahassee, Florida, USA; 2 Ohio State Biochemistry Program, The Ohio State University, Columbus, Ohio, USA

**Keywords:** CRISPR–Cas9, gene-editing enzyme, single-molecule FRET, conformational dynamics, induced-fit mechanism

## Abstract

*Streptococcus pyogenes* (*Sp*) Cas9 has been widely utilized to edit genomes across diverse species. To achieve high efficiency and specificity as a gene-editing enzyme, *Sp* Cas9 undergoes a series of sequential conformational changes during substrate binding and catalysis. Here, we employed single-molecule FRET techniques to investigate the effect of different KCl concentrations on conformational dynamics of *Sp* Cas9 in the presence or the absence of a single-guide RNA (sgRNA). In the absence of sgRNA and at low KCl concentrations (75 mM), apo Cas9 surprisingly exhibited two distinct conformations: a primary autoinhibited open conformation (apo Cas9 conformation [Cas9^apo^]) and a secondary sgRNA-bound-like conformation (Cas9^X^). Interestingly, increase in buffer KCl concentration led to a linear increase in the Cas9^X^ population and a corresponding decrease in the Cas9^apo^ population. In contrast, changes in KCl concentration exerted the opposite effects on the Cas9^X^ and Cas9^apo^ populations in the presence of sgRNA. Collectively, our findings by using KCl concentration as the probe suggest that Cas9 might employ a conformational sampling mechanism, in addition to the more common induced-fit mechanism established by us previously, for sgRNA binding.

Prokaryotes, including bacteria and archaea, possess a fascinating sequence-specific immune system, known as CRISPR and CRISPR-associated proteins (Cas), to defend against invading viruses and plasmids (1, 2). Among the three types of CRISPR–Cas systems, type II stands out for its simplicity by requiring only a dual-guide RNA and a Cas9 endonuclease. The dual-guide RNA is formed by a CRISPR RNA (crRNA) and a transactivating CRISPR RNA (tracrRNA). crRNA is a single-stranded RNA molecule (40–70 nucleotides) and forms a double-stranded RNA–DNA duplex (around 20 nucleotides) with the targeted DNA sequence. Single-stranded tracrRNA (80–100 nucleotides) uses some of its base pairs to form a double-stranded RNA region with a portion of the crRNA and induces conformational changes in Cas9, leading to its activation (3). For convenience, the crRNA and tracrRNA can be engineered to form a single-guide RNA (sgRNA), which possesses both functions of the dual-guide RNA (4). Presently, the most commonly used system for genome engineering is a type II CRISPR–Cas9 from the bacterium *Streptococcus pyogenes* (*Sp*) (2, 4). In 2023, Casgevy, a CRISPR–Cas9 gene-edited therapy, was approved for treating sickle cell disease in the United States, the United Kingdom, and the European Union.

*Sp* Cas9 possesses a bilobed architecture including an alpha-helical recognition (REC) lobe and a nuclease (NUC) lobe (5). NUC contains two nuclease (HNH and RuvC) domains and a C-terminal protospacer adjacent motif (PAM)–interacting domain (Fig. 1). HNH cleaves the on-target (transfer DNA [tDNA]) strand exactly three nucleotides away from the PAM, whereas RuvC initially cleaves the nontarget DNA (ntDNA) strand at multiple sites before trimming the cleavage ends in both the 3′→5′ and 5′→3′ directions (6) (Fig. 2). In 2018, we established a detailed kinetic mechanism of *Sp* Cas9 with the association of DNA to the binary complex of Cas9 and sgRNA being rate limiting during the first catalytic turnover and the extremely slow release of DNA products being the slowest step during the subsequent turnovers (7) (Fig. S1*A*). The conformational changes in *Sp* Cas9 are intricate and crucial for its function. For example, our previous kinetic and dynamic studies uncovered that HNH conformationally regulates the RuvC cleavage activity (7). Various crystal and cryo-EM structures of *Sp* Cas9 suggests that dynamic conformational changes in the enzyme facilitate nucleic acid binding (8, 9, 10, 11). In fact, the REC lobe (Fig. 1) undergoes major structural rearrangements with a 65 Å transition into close proximity to the HNH domain upon sgRNA binding, which is mediated by the bridge helix (residues 60–93) (9, 11, 12). Our recent article (7) shows that sgRNA binding by Cas9 induces the large conformational changes from an autoinhibited open conformation (apo Cas9 conformation [Cas9^apo]^) to the sgRNA-bound state conformation (Cas9–sgRNA) through two steps of the induced-fit mechanism Cas9^apo^ + sgRNA ⇌ Cas9^apo^–sgRNA ⇌ Cas9–sgRNA (the first two steps in Fig. S1*A*). Here, we employed single-molecule FRET (smFRET) assays to investigate whether the conformational transitions in *Sp* Cas9 can be altered by buffer ionic strength in the presence or the absence of sgRNA. Our results show that both apo- and sgRNA-bound forms likely coexisted in the absence of sgRNA, and their relative abundances were affected by both buffer ionic strength and sgRNA.

**Figure 1:**
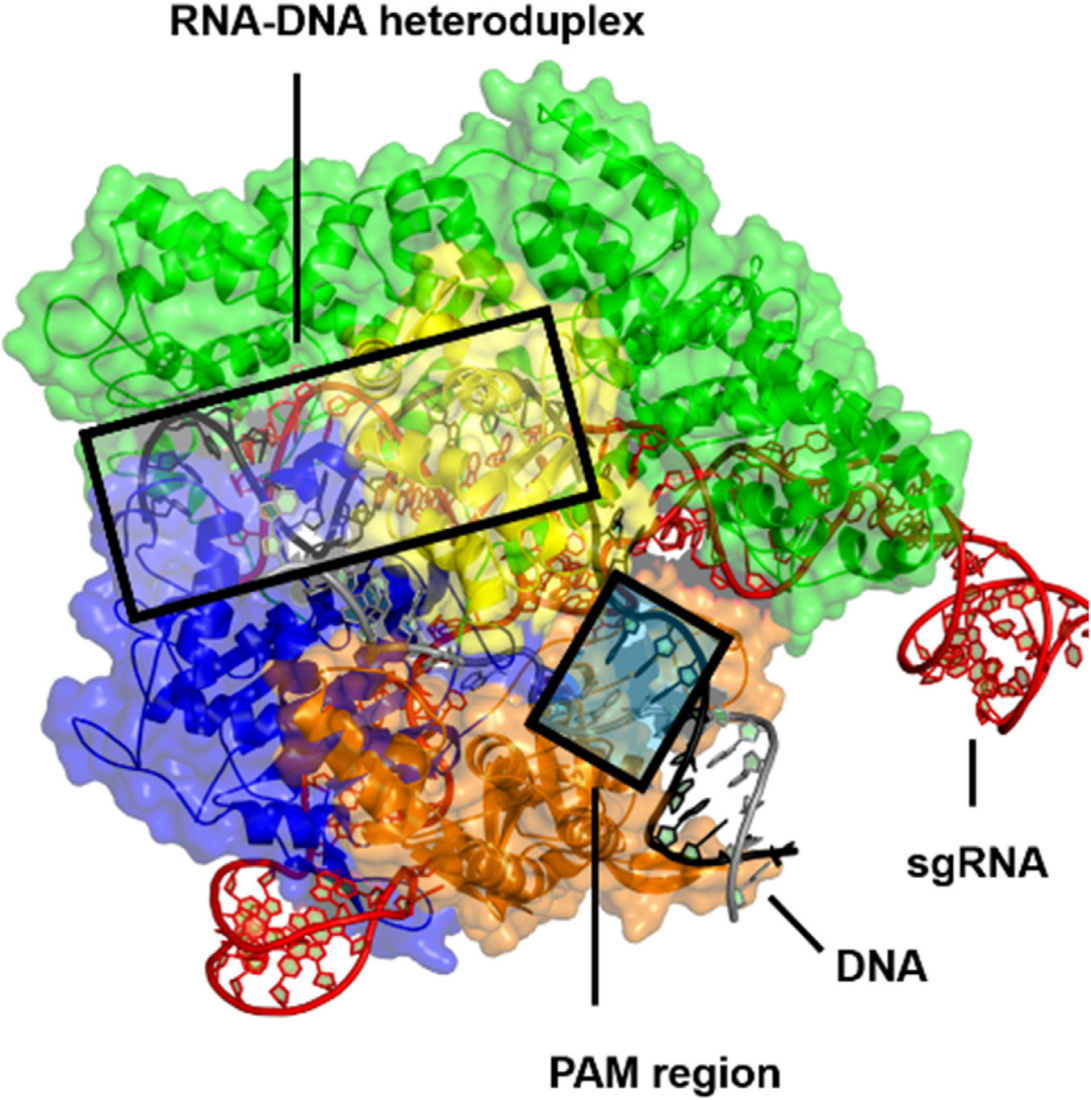
***Sp* Cas9 domain architecture (Protein Data Bank code:**59FR**).** Cas9 bound to sgRNA and on-target DNA (10). REC lobe, HNH, and RuvC nuclease domains and a C-terminal PAM-interacting (PIN) domain are shown in *green*, *yellow*, *blue*, and *orange*, respectively. Target and nontarget DNA strands are shown in *black* and *gray*, respectively. PAM, protospacer adjacent motif; REC, alpha-helical recognition; sgRNA, single-guide RNA; *Sp*, *Streptococcus pyogenes*.

**Figure 2:**
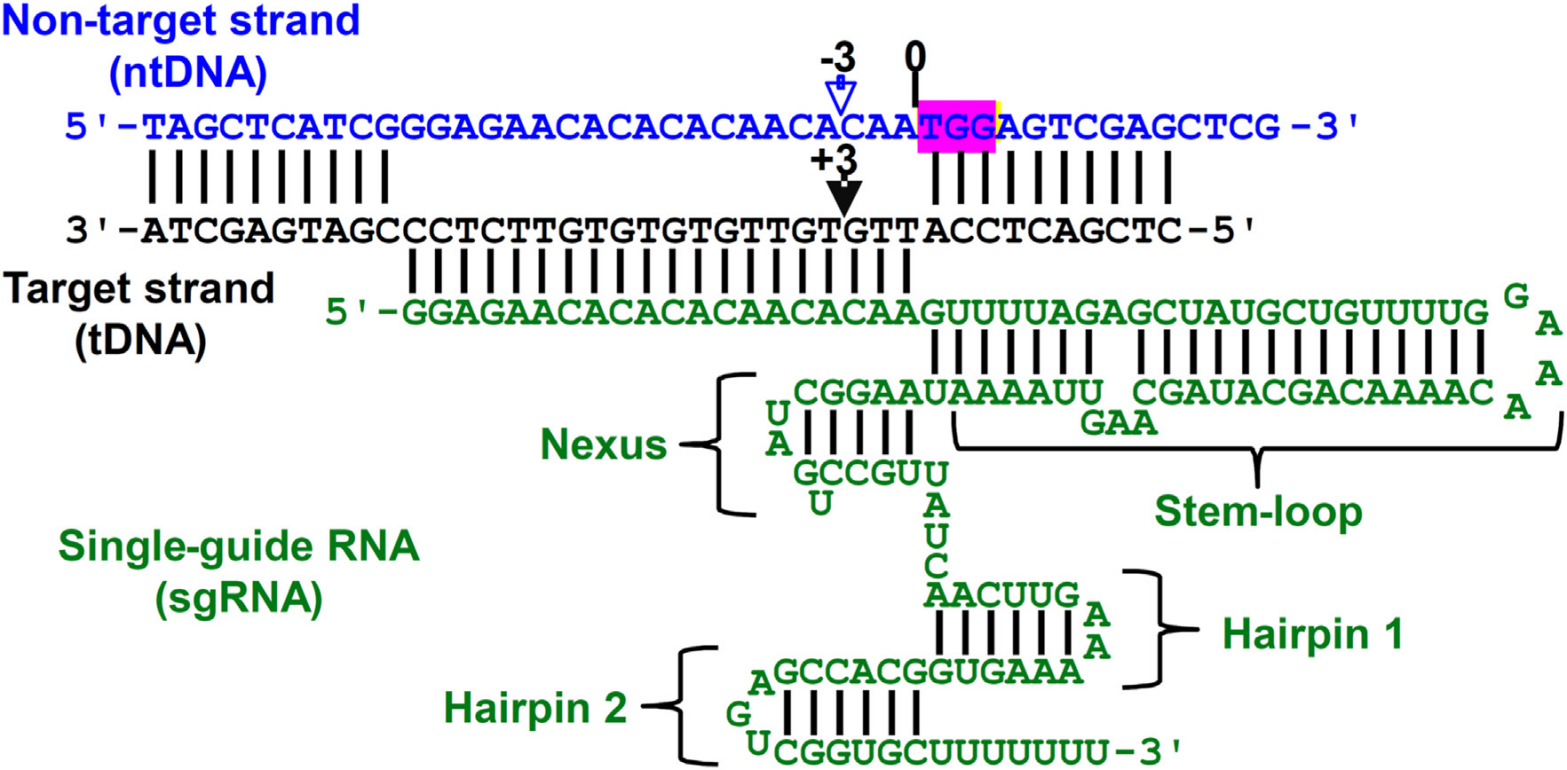
**sgRNA and DNA heteroduplex.** The PAM sequence on 44-mer (ntDNA) was highlighted in *pink*. Canonical cleavage sites are marked by *filled black* (HNH) and *open blue* (RuvC) *triangles*. ntDNA, nontarget DNA; PAM, protospacer adjacent motif; sgRNA, single-guide RNA.

## Results

### Conformational states of apo Cas9 identified at the single-molecule level

**Figure 3:**
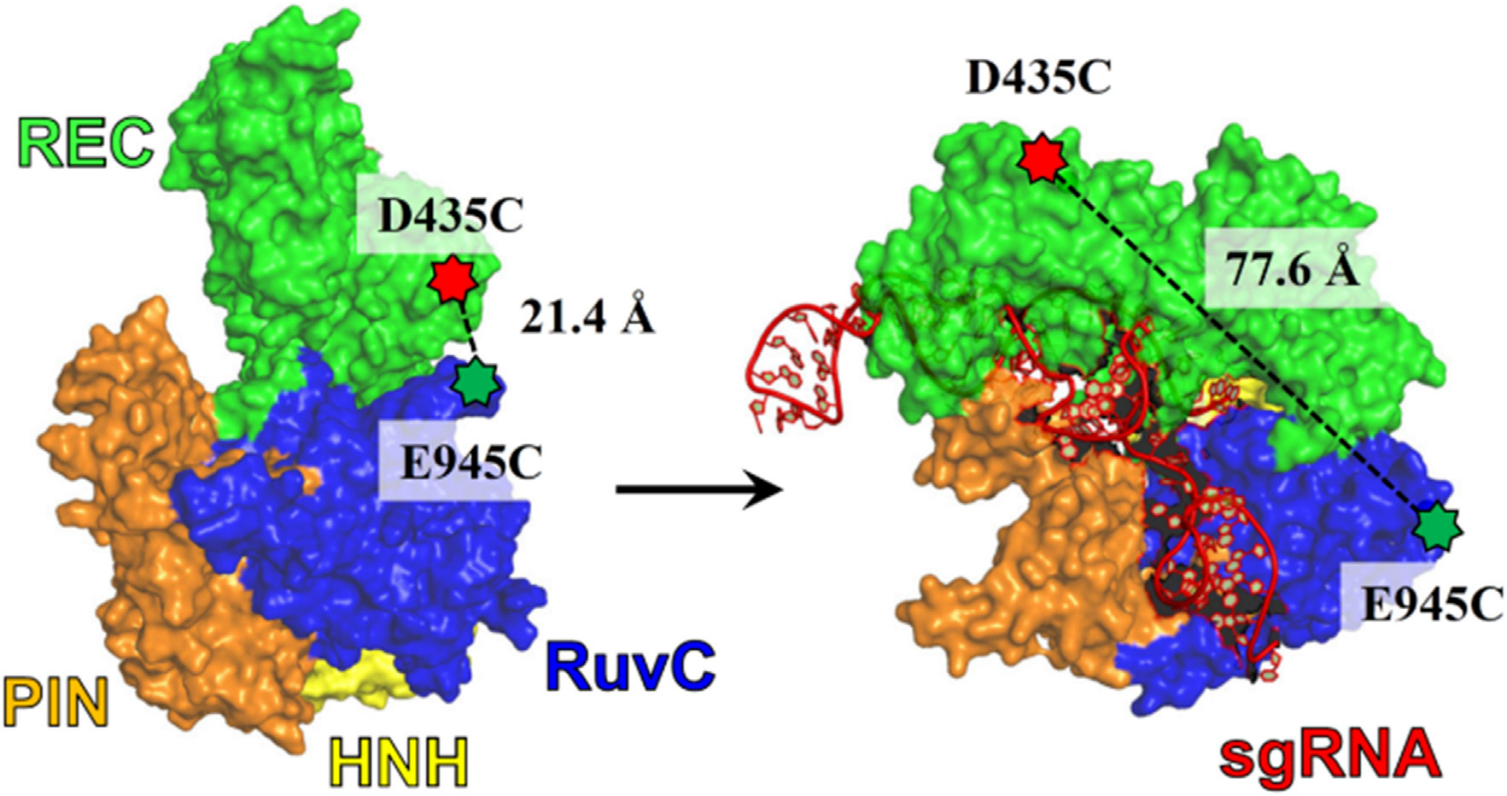
**FRET system (Cas9**^**FRET-R**^**)** (7) **to monitor Cas9 conformational change during transition from the apo state (Protein Data Bank code:**4CMP**) to the sgRNA-bound state (Protein Data Bank code:**4ZT0**).** Residues E945C (*green symbol*) and D435C (*red symbol*) of the biotinylated Cas9 mutant were labeled with Cy3 and Cy5, respectively. The distances between the residues were estimated from the indicated X-ray crystal structures. REC lobe, HNH, RuvC, and PIN domains are respectively shown in *green*, *yellow*, *blue*, and *orange*, whereas sgRNA is in *red*. Cas9^FRET-R^, an FRET system to monitor Cas9 conformational change during transition from the apo state to the sgRNA-bound state; PIN, PAM-interacting domain; REC, alpha-helical recognition; sgRNA, single-guide RNA.

**Figure 4:**
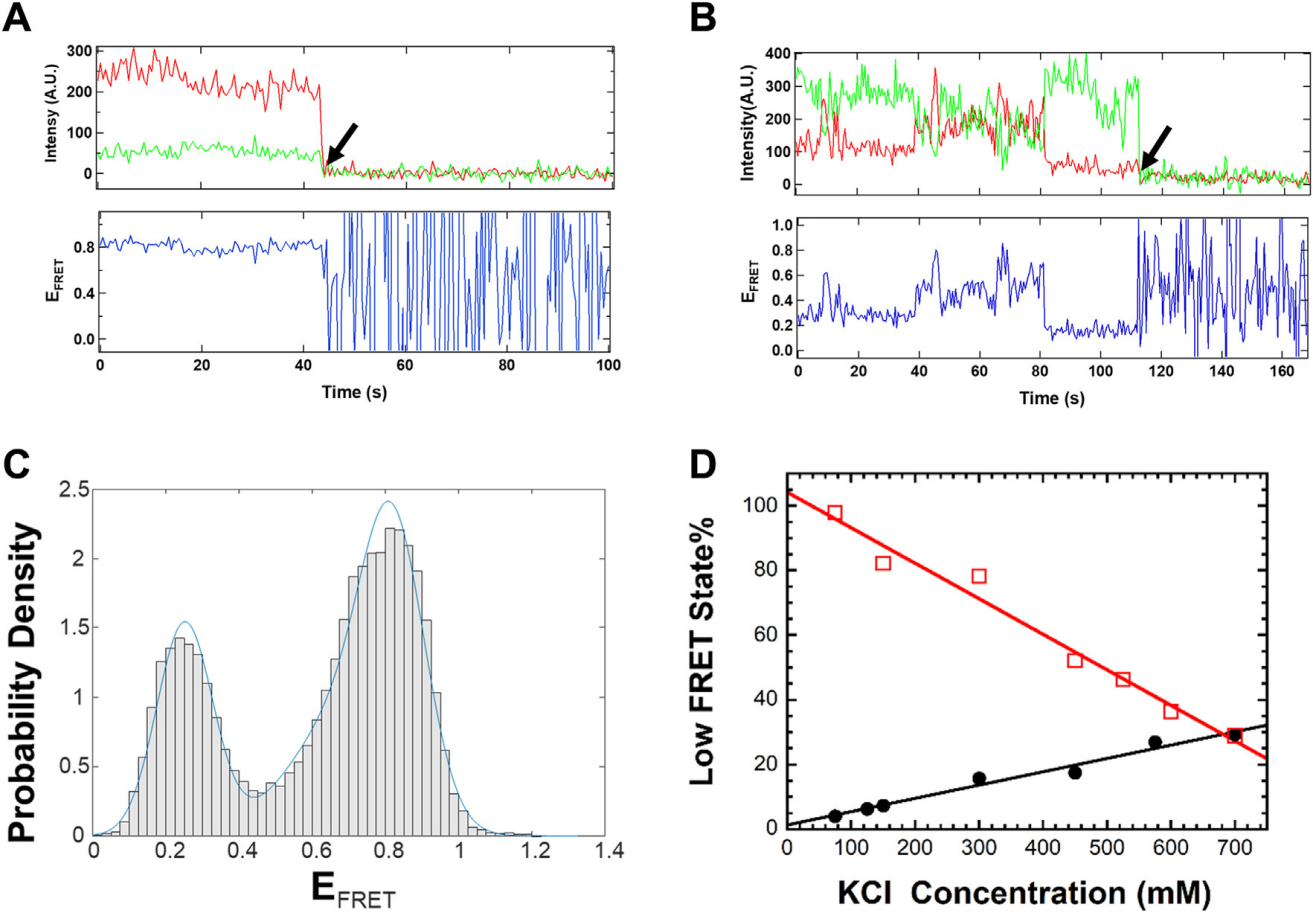
**Salt-induced conformational dynamics of *Sp* Cas9 at 25 °C.** Representative single-molecule trajectories of immobilized apo Cas9^FRET-R^ in the presence of 700 mM KCl depicting no transitions (*A*) and several transitions (*B*) between the high and low E_FRET_ (*right*) until donor photobleaching (*black arrow*). Donor (*green*) and acceptor (*red*) intensities were used to calculate E_FRET_ (*blue*). *C*, population distribution histogram from >300 single-molecule trajectories of immobilized apo Cas9^FRET-R^ at 700 mM KCl. The population distribution histogram was fit to a sum of Gaussians function (*blue line*) in MATLAB to extract the low-FRET and high-FRET state populations with the E_FRET_ peak positions at 0.25 and 0.80, respectively. *D*, percentage of the low-FRET state of Cas9 *versus* KCl concentration in the presence () or absence (•) of 20 nM sgRNA. The data were fit to a linear equation of y = 1.40 + 0.0411x with an *R*-factor of 0.986 in the absence (•) of 20 nM sgRNA and y = 104 − 0.110x with an *R*-factor of 0.988 in the presence () of 20 nM sgRNA. Cas9^FRET-R^, an FRET system to monitor Cas9 conformational change during transition from the apo state to the sgRNA-bound state; *Sp*, *Streptococcus pyogenes*.

As described previously (7), *Sp* Cas9 was expressed in *Escherichia coli* and purified through column chromatography (see the *Experimental procedures* section). To investigate conformational states of purified apo Cas9 (Fig. S2), we performed smFRET experiments (see the *Experimental procedures* section) by using our previously established FRET system, Cas9^FRET-R^ (Fig. 3), which is capable of monitoring donor–acceptor distance changes associated with Cas9 binding to sgRNA (7). Cas9^FRET-R^ possesses only two cysteine residues (E945C and D435C), which were labeled with Cy3 and Cy5 to form an FRET pair, and a C-terminal 15 amino acid residue AviTag, which was biotinylated at a specific lysine by BirA for surface immobilization during prism-based total internal reflection fluorescence imaging. The AviTag was fused with the unstructured C terminus of *Sp* Cas9 to prevent any disruption of protein conformational dynamics as a result of tagging and surface immobilization. Notably, the biotinylation and fluorescent labeling of Cas9 did not affect its nucleic acid–binding affinities and enzymatic activities in comparison to unmodified wildtype Cas9 (7). To carry out smFRET experiments, Cas9^FRET-R^ was surface immobilized in the imaging buffer containing different KCl concentrations and then excited at 532 nm for recording single-molecule movies at 2 frames/s. In the absence of sgRNA and at 700 mM KCl, we observed a majority of single-molecule trajectories exhibiting only the high FRET efficiencies (Fig. 4*A*), and the remaining single-molecule trajectories displaying multiple transitions between the high-FRET and low-FRET efficiencies (Fig. 4*B*). The population distribution histogram of >300 single-molecule trajectories at 700 mM KCl shows two peaks, and the bimodal distribution was fit to a sum of Gaussians function in MATLAB to obtain the population percentages of the low-FRET and high-FRET states among the total Cas9 conformation states to be 29.4% and 70.6%, respectively (Fig. 4*C*, Table 1).

**Table 1:** Distribution of two Cas9 FRET states in the absence of sgRNA as measured through single-molecule FRET at 25 °C

KCl concentration (mM)	High-FRET state (%)	Low-FRET state (%)
75	96.0	4.0
125	93.7	6.3
150	92.7	7.3
300	84.2	15.8
450	82.5	17.5
575	73.0	27.0
700	70.6	29.4

Previously, we did not consider any FRET events occurred in apo Cas9 with a threshold of E_FRET_ <0.2 in the imaging buffer containing 75 mM KCl (7). When we lowered the E_FRET_ threshold, we observed a population of molecules showing low-FRET efficiency and even several transitions between FRET efficiency states within the 208 single-molecule trajectories in our published studies (7). To further verify the result of the reanalysis, we collected 510 new single-molecule trajectories in the imaging buffer containing 75 mM KCl and discovered that the low-FRET and high-FRET conformational states comprised 4% and 96% of the total observed FRET events, respectively (Table 1, Fig. S3), and five of the total trajectories displayed E_FRET_ transitions between the states. Interestingly, as the concentration of KCl in the imaging buffer was increased, we observed more single-molecule trajectories with E_FRET_ transitions as shown in Fig. 4*B*, indicating that apo Cas9 became more conformationally dynamic under high salt conditions. Similarly, the percentages of the low-FRET and high-FRET conformational states at several KCl concentrations (Table 1) were determined from the fit of the bimodal population distribution histograms to a sum of Gaussians function (Fig. S3). The percentage of the low-FRET state was plotted against KCl concentration in the imaging buffer, and the plot (*black line*, Fig. 4*D*) was linear.

### Rates for the transitions between the low-FRET and high-FRET conformational states of apo Cas9 determined through dwell time measurements

**Figure 5:**
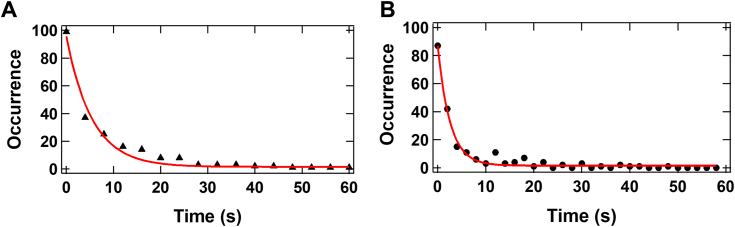
**Dwell time analysis of apo Cas9 conformational dynamics in the presence of 700 mM KCl at 25 °C.** The dwell times for the high-FRET and low-FRET states of immobilized Cas9^FRET-R^ were calculated from histograms of multiple single-molecule trajectories. The *red lines* represent fits to a single exponential equation to yield rate constants of 0.18 ± 0.01 s^−1^ for high → low FRET state transitions (*A*) and 0.39 ± 0.02 s^−1^ for low → high FRET state transitions (*B*).

Under a high KCl concentration, apo Cas9 became more conformationally dynamic, and multiple transitions between the high-FRET and low-FRET states of Cas9 were observed in each of many smFRET trajectories of Cas9^FRET-R^, for example, the transitions in Figure 4*B*. To further explore the conformational dynamics of apo Cas9, we determined the transition kinetics of the low-FRET and high-FRET conformational states *via* the dwell time analysis of our smFRET trajectories at 700 mM KCl. In brief, the duration of time each molecule spent at a particular FRET efficiency level before transitioning to a different level or photobleaching was determined. Dwell times of apo Cas9 in the low-FRET and high-FRET conformational states were binned into histograms, which were subsequently integrated, normalized, and inverted to yield survivor functions (Fig. 5). Subsequently, the survivor functions were fit to the single exponential decay equation to generate the high-FRET→low-FRET state transition rate (0.18 s^−1^, Fig. 5*A*) and the low-FRET→high-FRET state transition rate (0.39 s^−1^, Fig. 5*B*). Similarly, we employed dwell time analysis to yield the high-FRET→low-FRET state transition rate of 0.18 s^−1^ and the low-FRET→high-FRET state transition rate of 0.51 s^−1^ at 575 mM KCl (data not shown). Because of insufficient smFRET trajectories exhibiting conformational transitions at lower salt concentrations in the imaging buffer, we did not perform dwell time analysis of Cas9^FRET-R^ below 575 mM KCl.

### Effect of KCl concentration on conformational dynamics of *Sp* Cas9 in the presence of sgRNA at the single-molecule level

**Figure 6:**
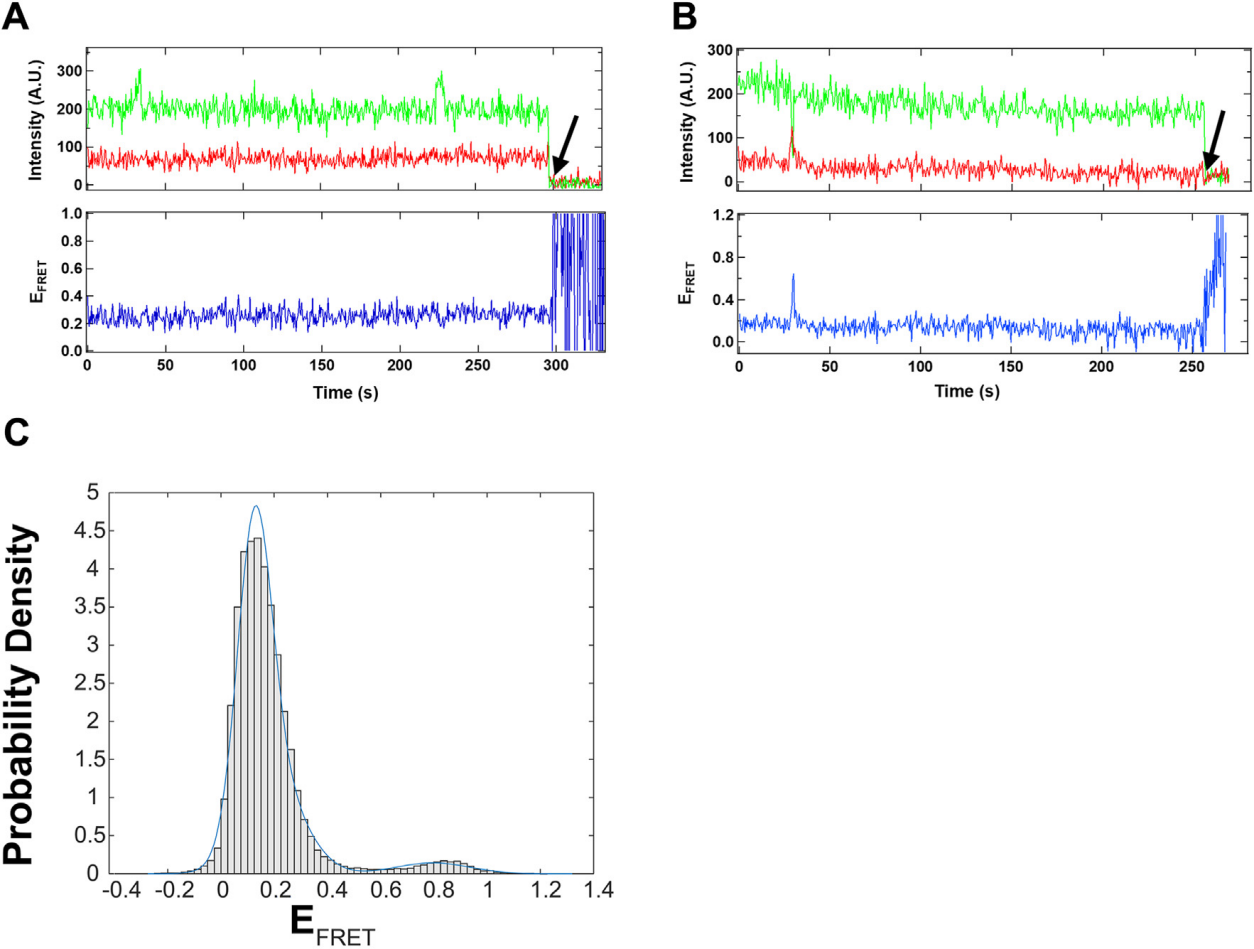
**Conformational dynamics of immobilized *Sp* Cas9 incubated with 75 mM KCl and 20 nM sgRNA at 25 °C.** Two representative single-molecule trajectories of immobilized Cas9^FRET-R^ without (*A*) and with (*B*) a transition between the high-FRET and low-FRET states until donor photobleaching (*black arrow*). Donor (*green*) and acceptor (*red*) intensities were used to calculate E_FRET_ (*blue*). *C*, the population distribution histogram from >300 single-molecule trajectories was fit to a sum of Gaussians function (*blue line*) in MATLAB to extract the low-FRET and high-FRET state populations with the E_FRET_ peak positions at 0.15 and 0.81, respectively. Cas9^FRET-R^, an FRET system to monitor Cas9 conformational change during transition from the apo state to the sgRNA-bound state; sgRNA, single-guide RNA; *Sp*, *Streptococcus pyogenes*.

We performed similar smFRET experiments to those described in Fig. 4 except that the imaging buffer also contained a fixed concentration of sgRNA (20 nM) besides varying KCl concentrations (see the *Experimental procedures* section). Given the reported high affinity of *Sp* Cas9 for sgRNA, with an apparent *K*_*d*_ of 10 pM (13), we anticipated that under relatively low salt concentrations, majority of immobilized Cas9 molecules in the imaging chamber would be predominantly bound by excess sgRNA. Consistently, in the presence of 75 mM KCl and 20 nM sgRNA, the immobilized Cas9^FRET-R^ molecules were mostly in their low-FRET state without any transitions, for example, the trajectory in Figure 6*A*. Only a small number of low-FRET to high-FRET transitions were observed, for example, one transition in Figure 6*B*. At 75 mM KCl, the population distribution histograms of Cas9 with (Fig. 6*C*) or without (Fig. S3) 20 nM sgRNA show two peaks with similar peak E_FRET_ values (0.15 and 0.84) but with an opposite dominant peak. In contrast, the histograms at 700 mM KCl with (Fig. S4) or without (Fig. 4*C*) 20 nM sgRNA resemble each other. Similarly, each of the population distribution histograms with 20 nM sgRNA was fit to a sum of Gaussians function in MATLAB to yield the percentages of the low-FRET and high-FRET state populations (Table 2). In the presence of 20 nM sgRNA, the low-FRET state dominated at low buffer salt, becoming subordinate at high buffer salt (Table 2). Interestingly, the plot of the percentage of the low-FRET state *versus* KCl concentration is linear (*red line*, Fig. 4*D*), and the two lines with and without 20 nM sgRNA intersect at ∼700 mM KCl (Fig. 4*D*). This means that the presence or absence of 20 nM sgRNA had no effect on the conformational states of Cas9 when the imaging buffer contained 700 mM KCl.

**Table 2:** Distribution of two Cas9 FRET states in the presence of 20 nM sgRNA as determined through single-molecule FRET at 25 °C

KCl concentration (mM)	High-FRET state (%)	Low-FRET state (%)
75	2.0	98.0
150	17.8	82.2
300	21.8	78.2
450	47.9	52.1
525	53.6	46.4
600	63.6	36.4
700	71.0	29.0

### Impact of KCl concentration on DNA cleavage activities of Cas9

**Figure 7:**
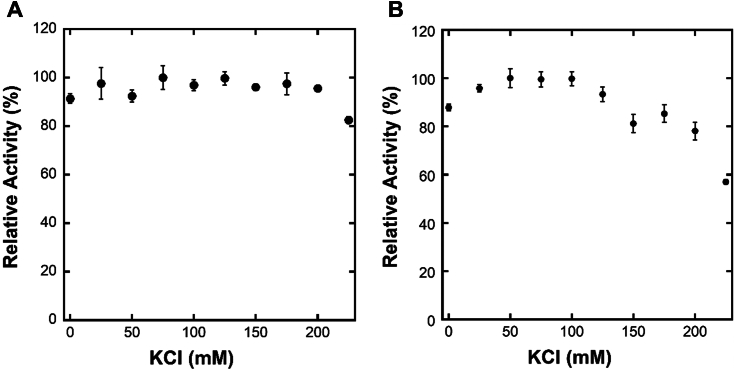
**Relative DNA cleavage activity of HNH (*A*) and RuvC (*B*) *versus* KCl concentrations at 37 °C.** The preincubated Cas9–sgRNA–DNA complex (10 nM) was mixed with MgCl_2_ (6 mM) in the reaction buffer containing 0 to 225 mM KCl to initiate DNA cleavage reaction for 15 s at 37 °C. The relative cleavage activity of HNH or RuvC, calculated as the percentage of the cleavage product amount over the total transfer DNA (tDNA) or nontarget DNA (ntDNA), from triplicate experiments was plotted against KCl concentration. sgRNA, single-guide RNA.

To check if the enzymatic activities of *Sp* Cas9 were affected by high salt concentrations, we performed DNA cleavage assays in the reaction buffer containing different KCl concentrations (see the *Experimental procedures* section). Reactions were stopped after 15 s and analyzed. Interestingly, the nuclease activities of HNH and RuvC were insignificantly affected by KCl concentrations up to 200 mM and 175 mM, respectively but decreased dramatically at higher concentrations (Fig. 7). High KCl concentrations likely reduced the nuclease activities of Cas9 by weakening the affinities of Cas9 for both sgRNA and DNA. This is because high salt concentrations can shield the charges on proteins, such as Cas9, and the nucleic acids, disrupting the electrostatic interactions that stabilize their complexes (14, 15). In addition, high salt can disrupt the hydration shells around the proteins including Cas9 and the nucleic acids, further compromising the stability of their complexes, such as Cas9–sgRNA and Cas9–sgRNA–DNA (14, 15).

## Discussion

### Detection of a previously unidentified conformation of apo Cas9 at the single-molecule level

*Sp* Cas9, a relatively large protein (∼158.4 kDa), contains the REC and NUC lobes in a single polypeptide chain (Fig. 1) and undergoes a series of conformational changes during sequential binding of sgRNA and DNA and subsequent tDNA and ntDNA cleavage by its HNH and RuvC nuclease activities, respectively (Fig. S1*A*) (7, 13). Here, we employed KCl concentration as a probe to investigate *Sp* Cas9 protein dynamics at the single-molecule level. Our smFRET analysis identified two distinct conformational states for apo Cas9: a high-FRET state and a low-FRET state (Figs. 4*C* and S3). To confirm that these states were not because of protein oligomerization, we performed native gel electrophoresis and observed a single band at ∼160 kDa (data not shown). Furthermore, we did not observe any aggregated apo Cas9 on the quartz slide surface during our smFRET experiments, which would appear with higher intensity than individually immobilized apo Cas9 molecules. These results indicate that the two conformational states arose from intrinsic properties of the monomeric apo Cas9. Notably, the percentage of the low-FRET state increased linearly with the concentration of KCl in the imaging buffer (Table 1 and Fig. 4*D*). The observed high-FRET state, termed Cas9^apo^, likely corresponds to the autoinhibited open conformation of apo Cas9 depicted in the *left structure* of Fig. 3. In this conformation, the donor and acceptor fluorophores are closer together, leading to a higher FRET efficiency. Conversely, the low-FRET state, referred to as Cas9^x^, is likely associated with a more compact structure, similar to the *right structure* in Fig. 3, with a longer distance between the donor and acceptor fluorophores. While the exact structure of Cas9^x^ remains unclear, it may resemble the sgRNA-bound conformation of Cas9 (Fig. 3, *right structure*) but without sgRNA (Fig. S5). Interestingly, Gaussian-accelerated molecular dynamics simulations predict that Cas9 adopts an intermediate conformation during its transition from Cas9^apo^ to sgRNA-bound state (16). The intermediate conformation, which is primed for RNA binding as the arginine-rich helix connecting the REC and NUC lobes of Cas9 becomes exposed to the solvent (16), may be related to Cas9^x^. Further structural investigation of apo Cas9 using techniques like cryo-electron microscopy or X-ray crystallography is needed to determine the exact structure of Cas9^x^ (see later).

### Establishment of the minimal kinetic mechanism for *Sp* Cas9 binding to sgRNA

**Figure 8:**
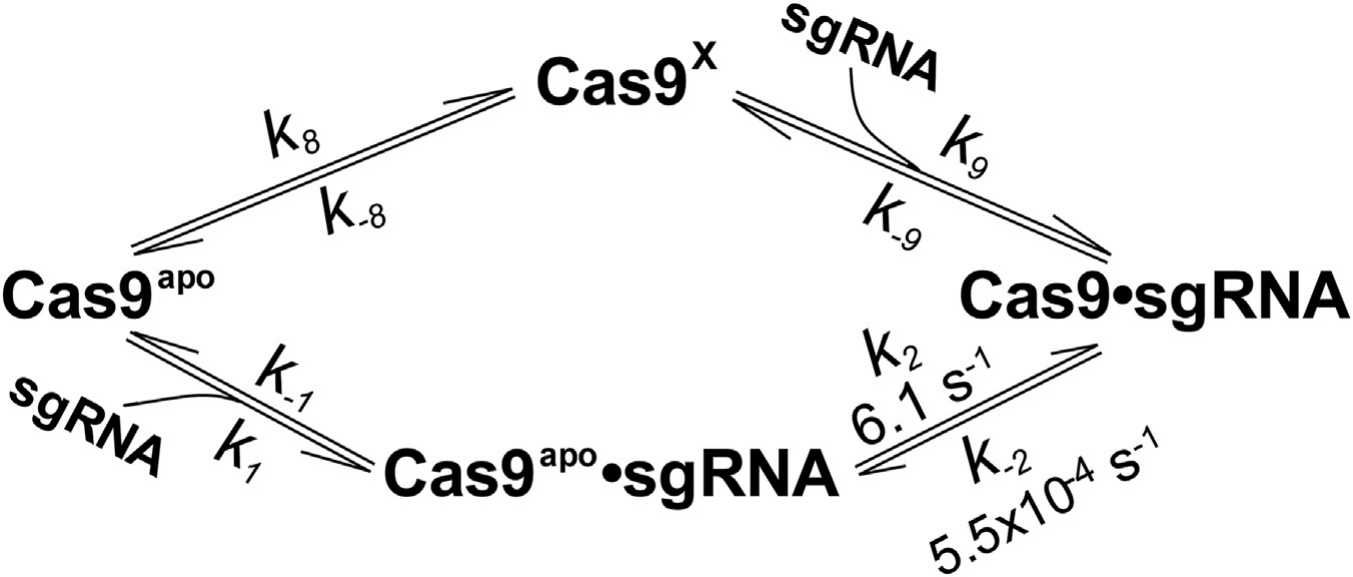
**Competing pathways for *Sp* Cas9 binding to sgRNA.** Cas9^apo^, Cas9^X^, and Cas9 denote the primary population of apo Cas9, the secondary population of apo Cas9, and sgRNA-bound Cas9, respectively. The *k*_2_ and *k*_-2_ values were previously determined by us *via* stopped-flow FRET assays at 125 mM KCl and 37 °C (7). Cas9^apo^, apo Cas9 conformation; sgRNA, single-guide RNA; *Sp*, *Streptococcus pyogenes*.

Structural analysis by other groups (see structure movie (13)) reveals that Cas9 in its sgRNA-bound conformation (*right structure*, Fig. 3) possesses a large cleft (Fig. S5), which can be snugly fit by sgRNA in a suitable conformation. If Cas9^x^ is in a structure as in Fig. S5 (see aforementioned), Cas9^x^ can be accessed and directly bound by sgRNA to form the Cas9–sgRNA complex. In other words, apo Cas9, like many other enzymes (17), may also employ the conformational sampling mechanism to bind sgRNA, that is, Cas9^apo^ ⇌ Cas9^x^ + sgRNA ⇌ Cas9–sgRNA (Fig. 8), besides the induced-fit mechanism, that is, Cas9^apo^ + sgRNA ⇌ Cas9^apo^–sgRNA ⇌ Cas9–sgRNA (Fig. S1*A*), previously hypothesized by structural biologists (13, 18) and established by us *via* both stopped-flow FRET and smFRET assays (7). For the conformational sampling mechanism to occur, it is possible that sgRNA may have to adjust its conformation to fit to the sgRNA-binding cleft of Cas9^x^. This possibility can occur when buffer ionic strength is reasonably high. Taking together, we propose an expanded kinetic mechanism for *Sp* Cas9 (Fig. S1*B*) with the addition of the competing pathways for sgRNA binding to Cas9^apo^ to form the Cas9–sgRNA complex (Fig. 8). The expanded kinetic mechanism contains more elementary steps than other minimal kinetic mechanisms proposed for *Sp* Cas9 in the literature (7, 19).

To determine the microscopic rate constants for the conformational changes of Cas9^apo^ ⇌ Cas9^x^ (Fig. 8), we performed dwell time analysis of the transitions between the high-FRET and low-FRET states of apo Cas9 at 25 °C. At 700 mM KCl, the dwell time analysis yielded the forward (*k*_8_ = 0.18 s^−1^) and reverse (*k*_-8_ = 0.39 s^−1^) rates (Fig. 5), leading to a calculated forward equilibrium constant (*K*_eq_ = *k*_8_/*k*_-8_ = 0.46). Similarly, *k*_8_ and *k*_-8_ were also determined to be 0.18 and 0.51 s^−1^ at 575 mM KCl, respectively, resulting in *K*_eq_ = 0.35. The slightly higher *K*_eq_ value at 700 mM KCl than at 575 mM KCl is due to the smaller *k*_-8_ value at higher buffer ionic strength, resulting in more Cas9^x^ formation. The dependence of *K*_eq_ on buffer ionic strength (16, 20, 21, 22, 23, 24) further indicates that important electrostatic interactions in apo Cas9 (Fig. S6) significantly influence the conformational changes of Cas9^apo^ ⇌ Cas9^x^. Furthermore, the percentage of the low-FRET state (=[*k*_8_/(*k*_8_+ *k*_-8_)] x 100%) was calculated to be 31.6% and 26.1% at 700 and 575 mM, respectively. These calculated low-FRET state values are comparable to 29.4% and 27.0% determined from the population distribution histogram analysis (Table 1), which validates the estimated *k*_8_ and *k*_-8_ values at 700 and 575 mM KCl. Notably, *k*_8_ and *k*_-8_ values were not determined at low KCl concentrations, for example, 125 mM, because of insufficient transitions between the high-FRET and low-FRET states in the smFRET trajectories. Interestingly, the addition of 20 nM sgRNA to apo Cas9 at 700 mM KCl caused negligible impact on the *k*_8_ and *k*_-8_ values (data not shown). Considering that ∼29% of the total Cas9 population was Cas9–sgRNA and possibly Cas9^x^ while the remaining 71% of Cas9 was Cas9^apo^ and possibly Cas9^apo^–sgRNA in the presence of 20 nM sgRNA and 700 mM KCl (Table 2), the negligible impact of the presence and absence of sgRNA on *k*_8_ and *k*_-8_ can be attributed to similar Cas9 conformational dynamics in Cas9^apo^ ⇌ Cas9^x^ and Cas9^apo^–sgRNA ⇌ Cas9–sgRNA in Fig. 8, whereas the steps of Cas9^apo^ ⇌ Cas9^apo^–sgRNA and Cas9^x^ ⇌ Cas9–sgRNA (Fig. 8) were not expected to generate FRET signals. It is also possible that the tight binary complex Cas9–sgRNA completely dissociated at 700 mM KCl. We are currently exploring this possibility through additional experiments.

### Modulation of conformationally dynamics of *Sp* Cas9 by buffer ionic strength

Notably, the interface between the REC and NUC lobes in Cas9^apo^ contains quite a few interacting charged amino acid residues (11) (Fig. S6). These electrostatic interactions were gradually disrupted by increasing KCl concentrations in the buffer, leading to the formation of additional Cas9^x^ at higher buffer ionic strength (Tables 1 and 2). For example, the low-FRET state, that is, Cas9^x^, at 25 °C was only 4.0% at 75 mM KCl but was increased to 29.4% at 700 mM KCl (Table 1). Under physiological salt concentrations (≤500 mM) (20, 25, 26, 27), only <20% of the total apo Cas9 as extrapolated from Fig. 4*D* (*black line*) are expected to be in the conformation state Cas9^x^. This means that a majority of apo Cas9 molecules *in vivo* are in the conformational state Cas9^apo^ and ready to be bound by sgRNA to form the eventual binary complex Cas9–sgRNA, *via* the induced-fit mechanism (Fig. 8). Consistently, the addition of excess sgRNA to apo Cas9 at <170 mM KCl in the buffer led to the dominant formation of Cas9–sgRNA (*red line*, Fig. 4*D*) and stable nuclease activities of Cas9 (Fig. 7). When the buffer salt concentration was >170 mM, the Cas9–sgRNA percentage (*red line*, Fig. 4D) and the nuclease activities of Cas9 were both lowered significantly (Fig. 7). However, the HNH and RuvC activities were not eliminated under the high end of the physiologically relevant salt concentrations (Fig. 7). This is probably why *Sp* Cas9 has been successfully employed in gene editing in almost all cell types despite different cellular salt concentrations (28, 29, 30, 31).

Interestingly, the conversion from Cas9^apo^ to Cas9^x^ showed the linear dependence on buffer ionic strength (Fig. 4*D*), a common feature reflected by charge–charge interactions among amino acid residues in protein (16, 20, 21, 22, 23, 24), for example, those in Cas9^apo^ (Fig. S6). Similarly, *Sulfolobus solfataricus* PCNA, containing a heterotrimeric ring structure, has been previously found to sample the ring-open and ring-closed conformations at one of its subunit interfaces, which contains multiple charge–charge interactions, and the transition to the ring-open conformation is linearly modulated by buffer ionic strength (20). At low buffer ionic strength, the aforementioned electrostatic interactions in Cas9^apo^ (Fig. S6) were strong, and the primary population of apo Cas9 in solution was determined to be Cas9^apo^, that is, the high-FRET state (Table 1). Under high buffer ionic strength, the electrostatic interactions in Cas9^apo^ (Fig. S6) were disrupted, and apo Cas9 became conformationally more dynamic, leading to multiple observed transitions between the high- and low-FRET states of apo Cas9 (Fig. 4*B*). Consistently, the low-FRET peak, that is, the Cas9^x^ peak, shifted its maximum E^FRET^ value from ∼0.15 at 75 mM KCl (Fig. S3) to ∼0.25 at 700 mM KCl (Fig. 4*C*), whereas the high-FRET peak, that is, the Cas9^apo^ peak, has a constant maximum E^FRET^ value (∼0.84) in the same range of KCl concentrations (Figs. 4*C* and S3). These observations indicate that Cas9^x^ is more conformationally dynamic than Cas9^apo^, which is expected to facilitate the snug-fit by sgRNA considering that sgRNA may also undergo conformational changes under high buffer ionic strength (32). Moreover, our smFRET analysis suggests that Cas9–sgRNA is as conformationally dynamic as Cas9^x^. If so, conformational dynamics of Cas9–sgRNA should facilitate PAM site search and DNA binding by Cas9 during gene editing (7, 33). For the induced-fit mechanism (Fig. 8), sgRNA likely utilizes its negatively charged nucleotides to disrupt the electrostatic interactions in rigid Cas9^apo^ (Fig. S6) and form the Cas9–sgRNA complex.

## Conclusions

Using KCl concentration as the probe, we performed smFRET assays to investigate *Sp* Cas9 conformational dynamics in the presence or the absence of sgRNA. We identified two populations of apo Cas9 at the single-molecule level, suggesting the existence of a previously unidentified conformational state of apo Cas9 (Cas9^x^). The population of Cas9^x^ depended linearly on buffer ionic strength. Based on kinetic and dynamic evidence, we presented a new mechanism for Cas9 binding to sgRNA (Fig. 8) and thus expanded our previously proposed kinetic mechanism for *Sp* Cas9 (Fig. S1*B*).

## Experimental procedures

### Preparation of *Sp* Cas9 and its mutant as well as fluorescent labeling of the mutant

Wildtype *Sp* Cas9 (residues 1–1368) and its mutant containing both the four mutations (C80S, C574S, D435C, and E945C) as well as a C-terminal 15 amino acid residue AviTag were constructed, expressed, and purified as previously described by us (7). Specifically, the pMJ806 plasmid encoding wildtype Cas9 or its aforementioned mutant, fused to an N-terminal His_6_ tag followed by a maltose-binding protein tag and a tobacco etch virus protease cleavage site, was transformed into LOBSTR *E. coli* BL21(DE3)-RIL-competent cells and then plated in LB agar containing 50 μg/ml kanamycin and 50 μg/ml chloramphenicol. One colony from the agar plate was inoculated 100 ml of LB broth containing 40 μg/ml kanamycin and 40 μg/ml chloramphenicol, and the culture was shaken overnight at 200 rpm and 30 °C until the absorbance at 600 nm reached ∼1.5. The primary culture (80 ml) was used to inoculate 8 l of LB pretreated with 35 μg/ml kanamycin and 40 μg/ml chloramphenicol, and the secondary culture was shaken at 200 rpm and 37 °C until the absorbance at 600 nm reached ∼0.6. Subsequently, 0.5 mM IPTG was added to the culture to induce protein expression for 10 hours at 200 rpm and 18 °C. The cells were harvested (4000 rpm, 20 minutes) and resuspended in buffer A (20 mM Tris–HCl, pH 8.0, 250 mM NaCl, 5 mM imidazole, and 10% glycerol). The resuspended cells were then lysed by passing through a French Press cell at 16,000 psi twice, and the resulting lysate was cleared by spinning in an ultracentrifuge (40,000 rpm, 45 min). Cleared lysate was incubated overnight at 4 °C with Ni Sepharose 6 Fast Flow resin (Cytiva), and the Cas9-bound nickel resin was then packed into a column. Bound proteins were washed with buffer A and eluted through a linear gradient of 0 to 100% buffer B (40 mM Tris–HCl, pH 8.0, 250 mM NaCl, 500 mM imidazole, and 10% glycerol). Tobacco etch virus protease (1 mg) was added to the pooled Cas9-containing fractions to cleave the N-terminal His_6_ and maltose-binding protein tags from the Cas9 fusion protein at 4 °C overnight. During the cleavage, the sample was simultaneously dialyzed against buffer C (40 mM Tris–HCl, pH 7.5, 150 mM KCl, 1 mM EDTA, 0.1% β-mercaptoethanol, and 10% glycerol). The dialyzed sample was applied to a HiTrap SP HP column (Cytiva), and the loaded column was then washed with buffer D (40 mM Tris–HCl, pH 7.5, 100 mM KCl, and 10% glycerol). The bound proteins were eluted *via* a linear gradient (100–1000 mM KCl) in buffer D. Fractions containing Cas9 and with the absorbance ratio of absorbance at 260 nm/absorbance at 280 nm less than 0.6 were pooled and dialyzed against buffer E (20 mM Hepes–KOH, pH 7.5, 500 mM KCl, 0.1% β-mercaptoethanol, and 10% glycerol) overnight. The dialyzed sample was centrifuged at 3900 rpm (∼3200*g*) for 5 min and injected onto a Superdex 200 increase 10/300 column (Cytiva) and eluted in buffer E. Fractions containing Cas9 with the absorbance at 260 nm/absorbance at 280 nm ratio less than 0.6 were pooled and concentrated to ∼50 μM. The concentrated Cas9 solution was finally dialyzed against the storage buffer (20 mM Hepes–KOH, pH 7.5, 500 mM KCl, 1 mM DTT, and 50% glycerol) overnight. Cas9 was purified to >95% purity based on staining SDS-PAGE gels with Coomassie Blue R-250 (Fig. S2). The concentration of the purified Cas9 was measured spectrophotometrically at 280 nm using the calculated extinction coefficient of 120,450 M^−1^cm^−1^. The absorbance at 260 nm/absorbance at _280_ nm ratio was below 0.6, indicating negligible nucleic acid contamination.

For smFRET measurements, the purified *Sp* Cas9 mutant (C80S, C574S, D435C, and E945C) with the C-terminal 15 amino acid AviTag was site-specific biotinylated with d-biotin and BirA, labeled with Cy3-and Cy5-maleimide (Lumiprobe Corporation), and purified through size-exclusion chromatography as outlined in our previous publication (7).

### Preparation of sgRNA and DNA substrates

By following our previously published procedures (6), sgRNA (Fig. 2) was prepared through *in vitro* transcription and purified *via* denaturing PAGE. Prior to each experiment, the sgRNA was folded in RNA annealing buffer (10 mM Tris, 50 mM NaCl, 1 mM EDTA, pH 7.5) by heating to 95 °C for 5 min and cooling slowly to 20 °C.

DNA oligonucleotides in Fig. 2 were purchased from Integrated DNA Technologies, Inc and individually purified by denaturing PAGE. The 40-mer and 44-mer (Fig. 1) were individually 5′-radiolabeled by incubation with [γ-^32^P]ATP (PerkinElmer) and T4 polynucleotide kinase (New England Biolabs) for 3 hours at 37 °C (34). Bio-Spin 6 size-exclusion columns (Bio-Rad) were used to remove unreacted [γ-^32^P]ATP. The radiolabeled 44-mer and 40-mer were mixed in a molar ratio of 1:1 and then annealed to each other by heating the mixture to 95 °C for 5 min followed by slow cooling to room temperature to generate the 40/44-mer DNA substrate (Fig. 2).

### Kinetic measurements and product and data analysis

All concentrations are reported as final concentrations after mixing. Unlabeled Cas9 (100 nM) and sgRNA (300 nM) were incubated for 30 min at 20 °C to form Cas9–sgRNA (100 nM). Next, the Cas9–sgRNA complex (100 nM) was incubated at 20 °C for 1 h with the doubly 5′-radiolabeled DNA substrate 40/44-mer (10 nM) to form Cas9–sgRNA–DNA (10 nM) in the absence of MgCl_2_. The Cas9–sgRNA–DNA complex (10 nM) was then rapidly mixed with MgCl_2_ (6 mM) in the reaction buffer A (20 mM Hepes [pH 7.5 at 37 °C], 1 mM EDTA, 1 mM DTT, and 10% glycerol) containing 0 to 225 mM KCl to initiate DNA cleavage reaction for 15 s at 37 °C. Reactions were terminated using quench solution with a final composition of 0.37 M EDTA, 20% formamide, 0.25% bromophenol blue, and 0.25% xylene cyanol. Denaturing PAGE (12% polyacrylamide, 8 M urea, and 1x TBE) was used to separate remaining DNA substrates and newly formed DNA cleavage products. Gels were scanned using a Typhoon RGB (GE Healthcare) and quantified by densitometry in ImageQuant (Molecular Dynamics). Notably, RuvC product formation was quantified as the sum of all ntDNA-cleavage products, whereas the only HNH cleavage product was 13-mer as observed by us previously (7). The relative cleavage activity of HNH or RuvC under a specific KCl concentration was calculated as the percentage of the cleavage product(s) over the sum of the remaining substrate (tDNA or ntDNA) and the cleavage product(s).

### smFRET measurements

smFRET measurements were performed on our custom-built prism-based total internal reflection fluorescence microscopy system as described previously (7, 35, 36, 37). Briefly, quartz microscope slides were functionalized and passivated before imaging chamber assembly and addition of NeutrAvidin (0.2mg/ml) (ThermoFisher). Biotinylated, apo Cas9 (100 pM) was then surface immobilized, and the imaging chamber was rinsed with T50 buffer (10mM Tris–HCl, pH 8.0, 50 mM KCl). The chamber was rinsed three times with the imaging buffer (35 mM Hepes, pH 7.5, 75–700 mM KCl, 2 mM Trolox, 1 mM EDTA, 1 mM DTT, 0.1 mg/ml bovine serum alnumin, 0.8 w/v d-glucose, 1 mg/ml glucose oxidase [Sigma], and 0.004 mg/ml catalase [Calbiochem]) with or without sgRNA (20 nM). Next, the Cy3- and Cy5-labeled Cas9 was excited at 532 nm, and single-molecule movies were recorded at 25 °C and 2 frames/s using an Andor iXon 897 EMCCD over several minutes. Movies were subsequently processed using IDL (ITT Visual Information Solutions) and custom MATLAB scripts (Center for the Physics of Living Cells, University of Illinois Urbana-Champaign) to map single molecules in the donor (Cy3) and acceptor (Cy5) emission channels. The resulting donor and acceptor fluorescence intensity trajectories were background corrected in MATLAB and used to calculate apparent FRET efficiency (*E*_*FRET*_) using Equation 1,

(1) $$E_{FRET}=\frac{I_{A}}{I_{D}+I_{A}}$$

where *I*_D_ and *I*_A_ are the donor and acceptor fluorescence intensities, respectively. Molecules demonstrating clear anticorrelated donor and acceptor signals were collected, and the FRET efficiencies were binned to generated FRET population distribution histograms. Signal following donor or acceptor photobleaching, which resulted in a zero-FRET efficiency state, was removed from each single-molecule trajectory. Each population distribution histogram was fit to a sum of Gaussian functions using MATLAB, and percent occupancy of FRET states was calculated as the total area under the individual Gaussian fits. Dwell time analysis was performed as previously described (36, 38). Briefly, the duration of each FRET event for the selected molecules was quantified using a thresholding analysis. The “low-FRET” state was limited by thresholds at FRET efficiencies from 0.2 to 0.5, and the “high-FRET” state was limited by thresholds at FRET efficiencies from 0.5 to 0.8. The resulting dwell times were compiled to generate survivor functions as previously described (36, 38) which were fit to a single exponential decay equation (Equation 2),

(2) $$f\left(t\right)=Ae^{-kt}$$

where *f*(*t*) is the fraction of molecules in the designated FRET state after time *t*, *A* is the amplitude of the function, and *k* is the decay rate constant associated with the designated FRET state.

## Data availability

Any additional information required to analyze the data reported in this article is available from corresponding author upon request.

## Supporting information

This article contains supporting information.

## Conflict of interest

The authors declare that they have no conflicts of interest with the contents of this article.
