# Supplementary material for: Conformational transitions of Streptococcus pyogenes Cas9 induced by salt and single-guide RNA binding
Source: J Biol Chem. 2024 Dec 21;301(2):108120. doi: 10.1016/j.jbc.2024.108120 (PMC11791316; doi:10.1016/j.jbc.2024.108120)
Supplement: Supporting Information [file mmc1.pdf]

Supporting Information

Conformational transitions of *Streptococcus pyogenes* Cas9 induced by salt and single-guide RNA binding

Yufan He[1], Nikita Zalenski[1], Anthony A. Stephenson[2], Austin T. Raper[2], Chiran Ghimire[1], and Zucai Suo[1,2,*]

[1]Department of Biomedical Sciences, College of Medicine, Florida State University, Tallahassee, FL 32306, USA

[2]Ohio State Biochemistry Program, The Ohio State University, Columbus, OH 43210

*To whom correspondence should be addressed: Zucai Suo, Department of Biomedical Sciences, College of Medicine, Florida State University, Tallahassee, FL 32306, USA; Tel.: (850) 645-2501; E-mail: zucai.suo@med.fsu.edu

Figures S1 to S6

A

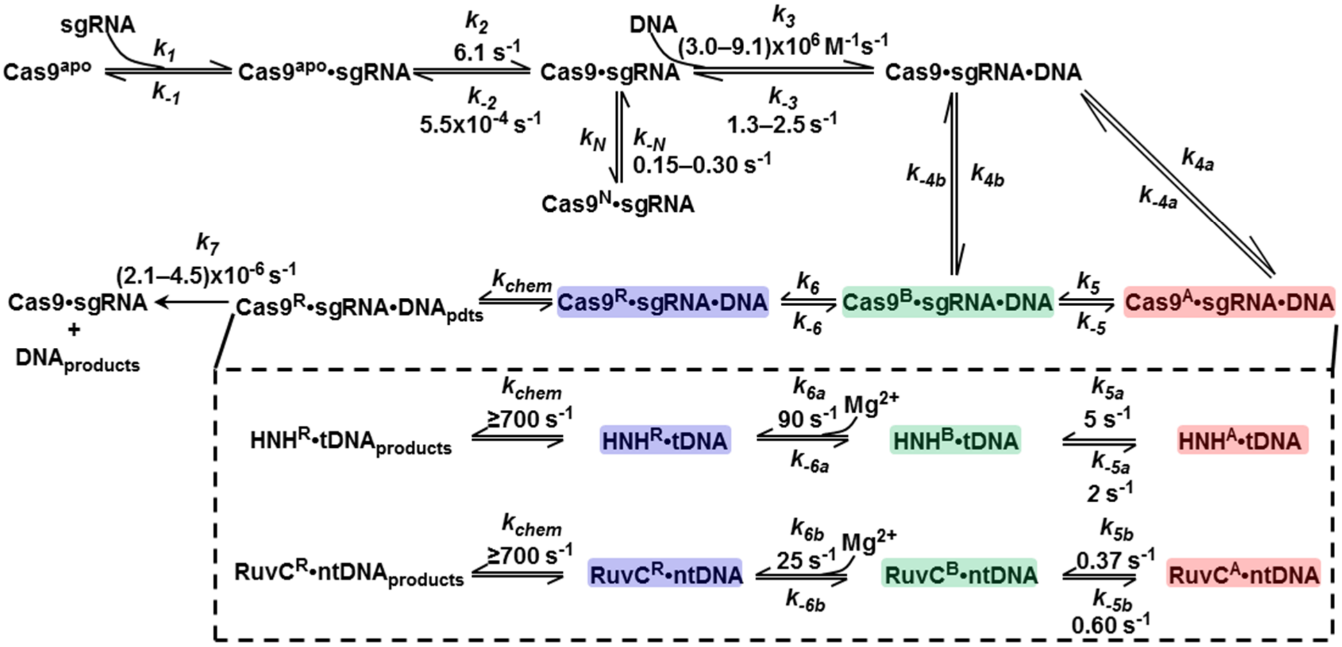

B

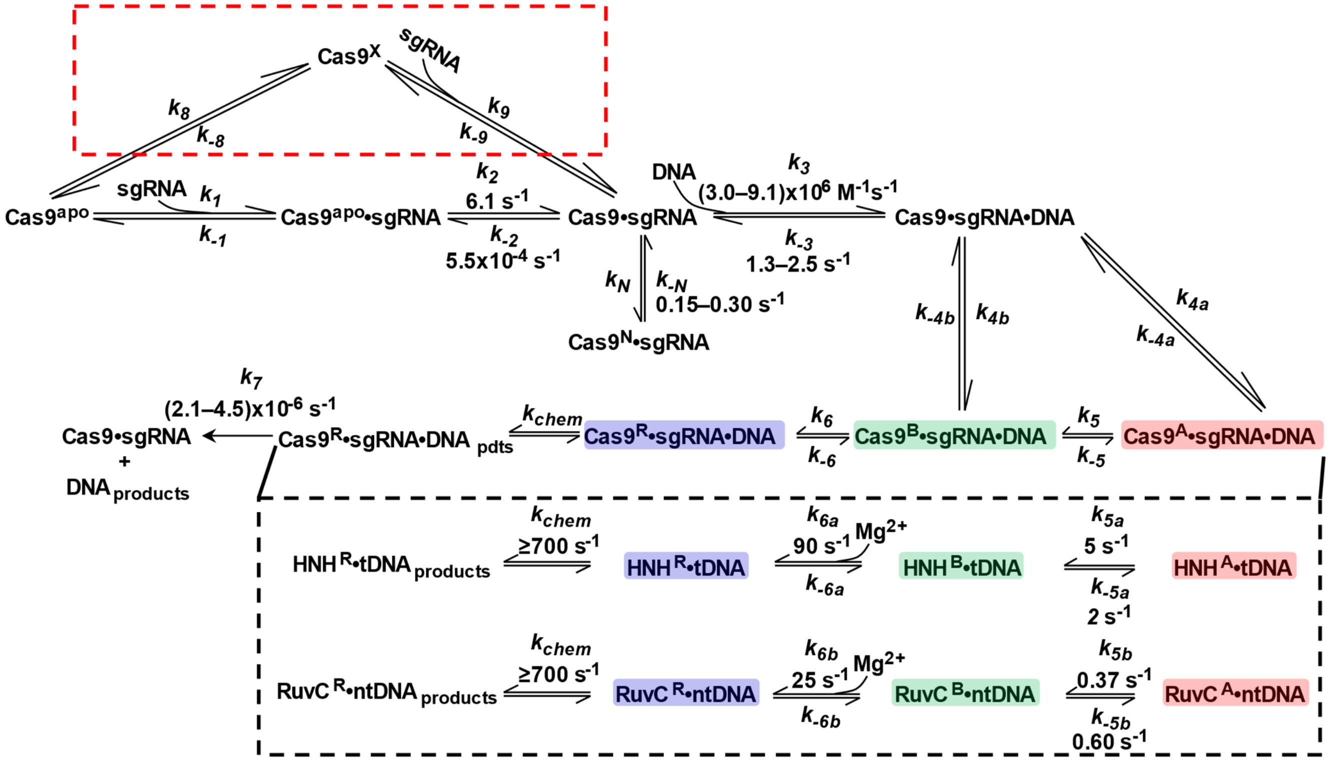

Figure S1. Minimal kinetic mechanism of Cas9 in the presence of Mg^{2+}. (A) Published kinetic mechanism by us previously (1). (B) Expanded kinetic mechanism based on this paper. The dashed black box depicts kinetic contribution of conformational heterogeneity of the HNH and

RuvC domains to DNA cleavage. The dashed red box highlights the conformational sampling pathway for sgRNA binding to Cas9 as elucidated in this paper. The depiction of Mg^{2+} at certain steps denotes the Mg^{2+} binding-coupled conformational changes of HNH and RuvC, rather than second-order metal ion binding.

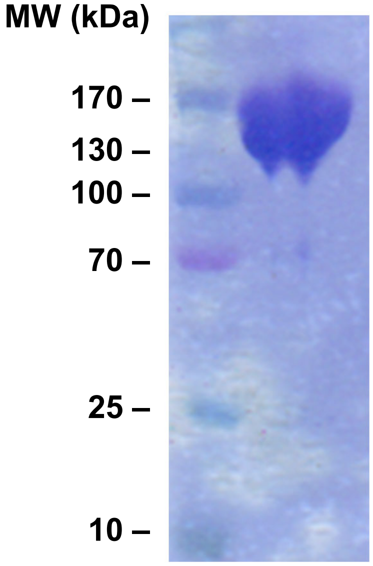

Figure S2. Purified Cas9 (right lane) was analyzed by SDS-PAGE. Standard protein markers (peqGOLD pre-stained protein marker IV, VWR) were shown on the left lane.

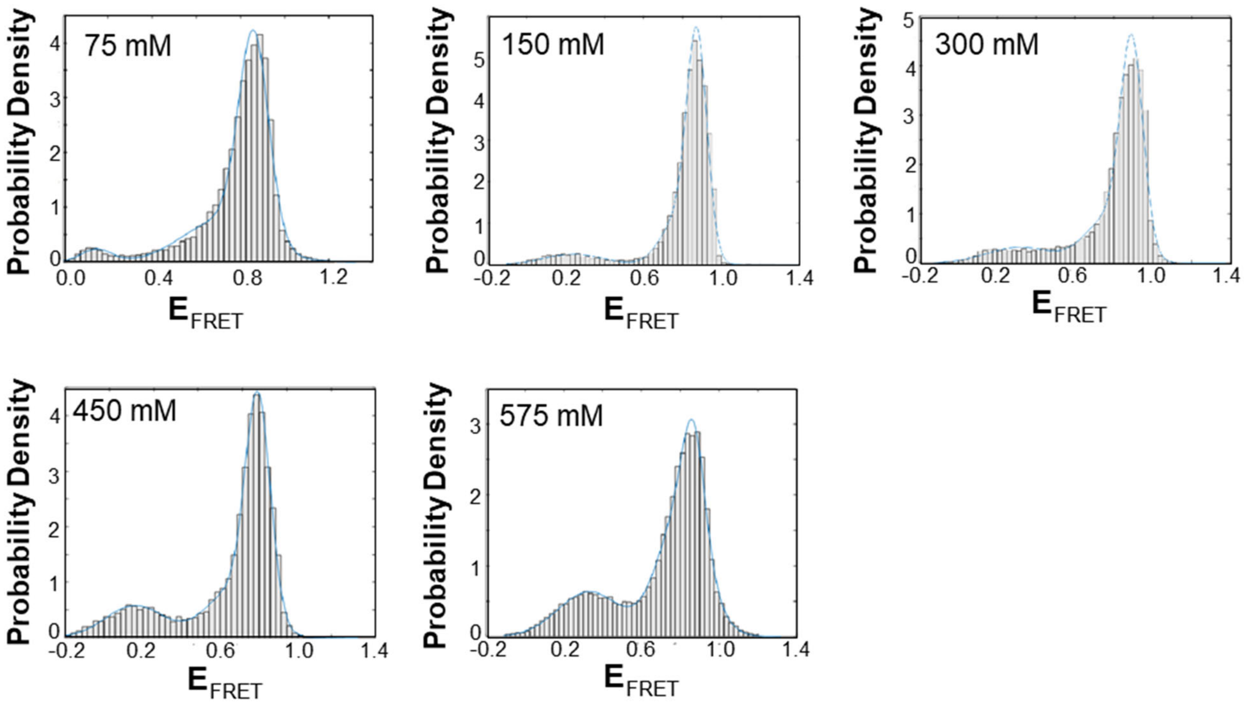

Figure S3. Population distribution histograms featuring FRET efficiencies (E_{FRET}) from >200 single-molecule trajectories of immobilized apo-Cas9^{FRET-R} incubated with indicated KCl concentrations at 25 °C. The bimodal population distribution histogram at an indicated KCl concentration was fit to a sum of Gaussians function (blue line) in MATLAB to extract the individual low-FRET and high-FRET state populations. The low-FRET and high-FRET E_{FRET} peak positions are respectively 0.11 and 0.84 at 75 mM KCl, 0.23 and 0.87 at 150 mM KCl, 0.27 and 0.87 at 300 mM KCl, 0.16 and 0.81 at 450 mM KCl, 0.23 and 0.85 at 575 mM KCl.

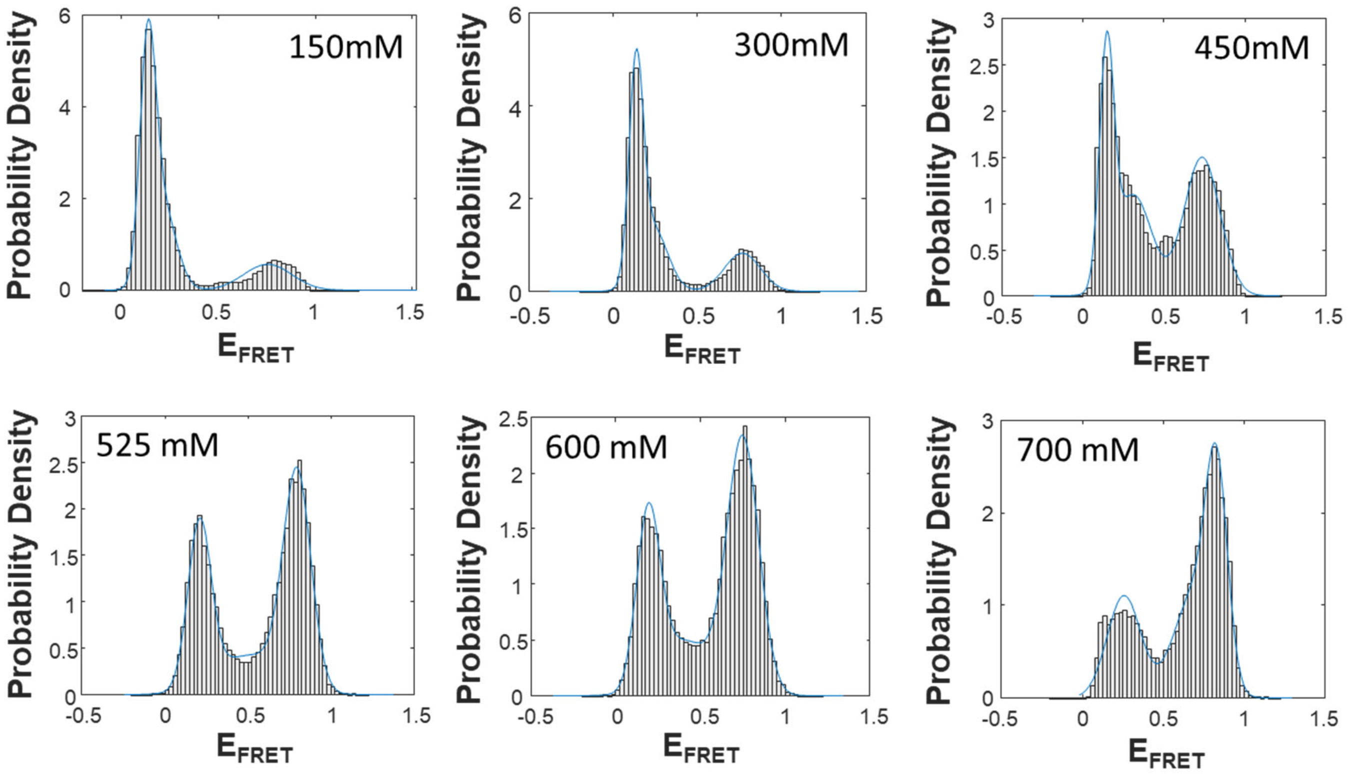

Figure S4. Population distribution histograms featuring FRET efficiencies (E_{FRET}) from single-molecule trajectories of immobilized apo-Cas9^{FRET-R} incubated with indicated KCl concentrations and 20 nM sgRNA at 25 °C. Each bimodal population distribution histogram was fit to a sum of Gaussians function (blue line) in MATLAB to extract the individual low-FRET and high-FRET state populations. The low-FRET and high-FRET E_{FRET} peak positions are respectively 0.16 and 0.75 at 150 mM KCl, 0.16 and 0.76 at 300 mM KCl, 0.15 and 0.74 at 450 mM KCl, 0.25 and 0.79 at 525 mM KCl, 0.23 and 0.75 at 600 mM KCl, 0.26 and 0.80 at 700 mM.

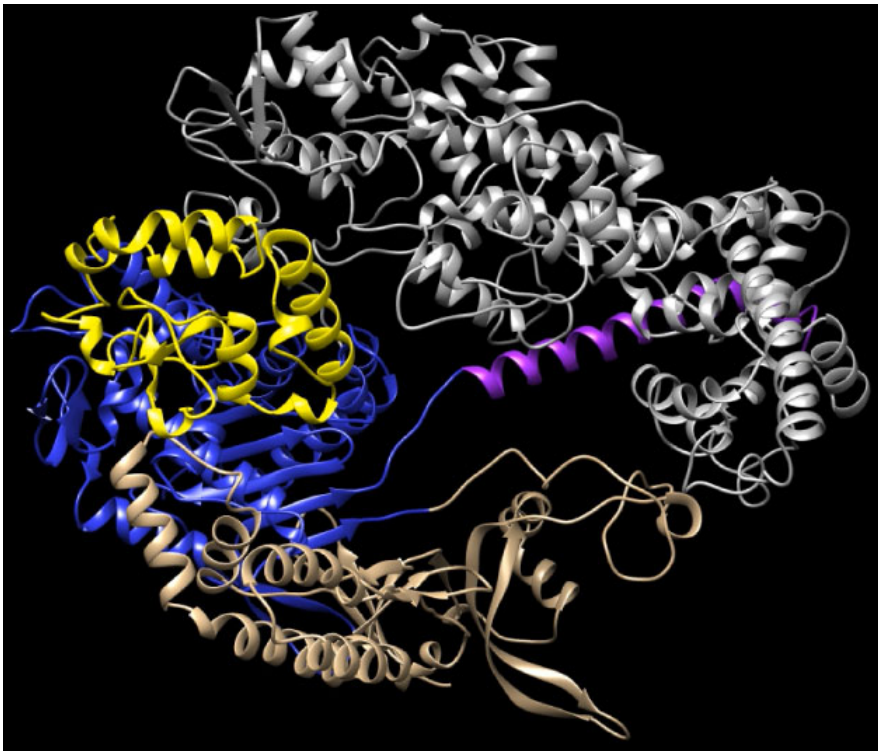

Figure S5. Potential domain structure of Cas9^{x}. This structure is the same as the Cas9 structure in the Cas9•sgRNA complex (PDB code: 4ZT0) (2) but with sgRNA hidden. REC lobe, HNH and RuvC nuclease domains, PIN domain, and bridge helix are shown in white, yellow, blue, beige, and purple, respectively.

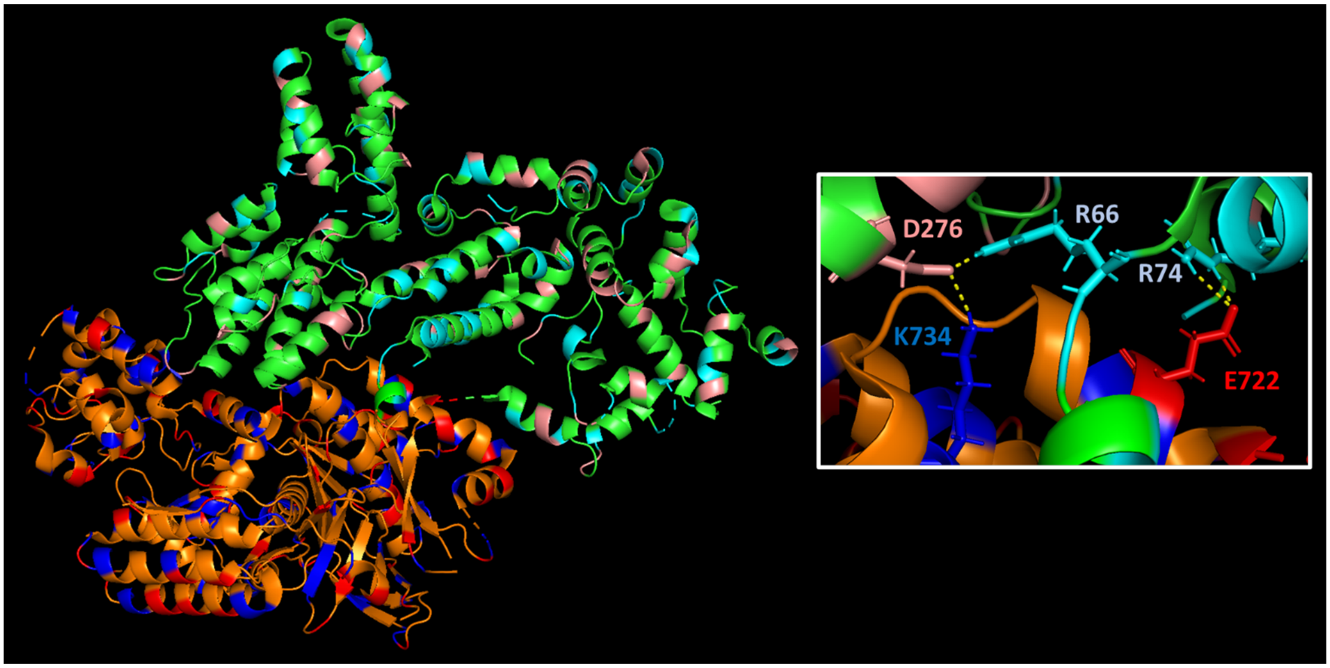

Figure S6. Interactions within the interface between the REC and NUC lobes in Cas9^{apo}. In the structure (PDB code: 4CMP), REC and NUC lobes are shown in green and orange respectively. Negatively and positively charged residues are shown in pink and cyan, respectively, in the REC lobe; red and blue, respectively, in the NUC lobe. A magnified view of the interface between the REC and NUC lobes is presented in the inset, with electrostatic interactions indicated by yellow dashed lines.

REFERENCES

1. Raper, A.T., Stephenson, A.A. and Suo, Z. (2018) Functional Insights Revealed by the Kinetic Mechanism of CRISPR/Cas9. *J Am Chem Soc*, **140**, 2971-2984.
2. Sternberg, S.H., LaFrance, B., Kaplan, M. and Doudna, J.A. (2015) Conformational control of DNA target cleavage by CRISPR-Cas9. *Nature*, **527**, 110-113.
